# Antimicrobial Effect of Cinnamaldehyde and *α*-Terpineol on Endodontic Biofilms of *Candida albicans* and *Enterococcus faecalis*

**DOI:** 10.1155/ijm/4769807

**Published:** 2025-05-13

**Authors:** Maria Heloísa de Souza Borges-Grisi, Arella Cristina Muniz Brito, Isis Morais Bezerra, Loyse Martorano-Fernandes, Yuri Wanderley Cavalcanti, Leopoldina de Fátima Dantas de Almeida

**Affiliations:** ^1^Department of Clinical and Social Dentistry, Federal University of Paraíba-UFPB, João Pessoa, Paraíba, Brazil; ^2^Department of Prosthodontics and Periodontology, Campinas State University-Unicamp, Piracicaba, São Paulo, Brazil

**Keywords:** antimicrobial agents, biofilms, biological products, root canal therapy

## Abstract

**Objectives:** The objective was to assess the antimicrobial effect of cinnamaldehyde and *α*-terpineol on mono-species and dual-species biofilms involved in endodontic infection.

**Materials and Methods:** The phytoconstituents were used at a concentration of 10 mg/mL. The biofilms of *Candida albicans* (ATCC 90028) and *Enterococcus faecalis* (ATCC 29212) were developed for 7 days and evaluated by metabolic capacity analysis using MTT, cell viability analysis by CFU/mL, and phospholipase activity. The RPMI 1640 medium was used as the negative control and sodium hypochlorite 2.5% and chlorhexidine 2% were used as positive controls. Data were analyzed by a Kruskal–Wallis test and stepwise with adjusted Bonferroni for nonnormal data and an ANOVA one-way test followed by Tukey's post hoc test for normal data (*α* = 5%).

**Results:** The cellular metabolism of the *C. albicans* and *E. faecalis* mono-species biofilms was reduced by cinnamaldehyde and *α*-terpineol (*p* < 0.05). For dual-species biofilm, only *α*-terpineol showed differences compared to the negative control (*p* < 0.05). The phytoconstituents showed an inhibitory effect on cell viability (CFU/mL) and phospholipase activity of biofilms, having an activity similar to sodium hypochlorite (*p* > 0.05).

**Conclusions:** The phytoconstituents cinnamaldehyde and *α*-terpineol, at a concentration of 10 mg/mL, had an inhibitory effect on mono-species and dual-species biofilms of *E. faecalis* and *C. albicans*.

## 1. Introduction

Endodontic infection is a polymicrobial disease primarily resulting from the progression of dental caries into the pulp tissue. Initially, the endodontic biofilm consists of aerobic and facultative anaerobic species. As the infection progresses, the biofilm undergoes ecological changes, increasing its virulence and resistance to clinical intervention [1, 2].

The complex anatomy of the root canal system and the interactions between various species of microorganisms complicate the eradication of the infection, leading to treatment failure [3]. *Enterococcus faecalis* and *Candida albicans* are frequently associated with persistent and refractory endodontic infections [4]. *E. faecalis*, a facultative anaerobic gram-positive bacterium, demonstrates high adaptability to hostile environments and is commonly found in chronic apical periodontitis [4]. *C. albicans*, the most prevalent fungal species in pulp-origin infections, can penetrate dentinal tubules and evade conventional antimicrobial treatments [5].

Root canal disinfection relies on chemical irrigants and intracanal medications with antimicrobial efficacy and biocompatibility [6]. Sodium hypochlorite (NaOCl) is widely used due to its antimicrobial activity and capacity to dissolve organic tissue; however, its cytotoxicity, instability, and negative effects on dentin integrity limit its application [7, 8]. Alternatively, 2% chlorhexidine digluconate (CHX) exhibits broad-spectrum antimicrobial activity and prolonged substantivity; however, it lacks the ability to dissolve organic tissue and does not eliminate endotoxins [7, 8].

Due to the adaptive resistance of endodontic biofilms to conventional antimicrobial agents, alternative therapeutic strategies are being actively investigated [9–11]. Phytoconstituents such as cinnamaldehyde and *α*-terpineol have shown promising anti-inflammatory and antimicrobial properties [12–14], but their effects on microorganisms associated with persistent endodontic infections are not elucidated in the literature.

Therefore, the objective of this study was to evaluate, in vitro, the antimicrobial effects of cinnamaldehyde and *α*-terpineol against *E. faecalis*and *C. albicans*, microorganisms related to persistent endodontic infections. The general hypothesis of this work is that cinnamaldehyde and *α*-terpineol exhibit antimicrobial action on *C. albicans* and *E. faecalis* mono-species and dual-species biofilms.

## 2. Materials and Methods

### 2.1. General Study Design and Sample Number

An *in vitro*, controlled, and blinded study was performed. The laboratory assays were divided into microbiological tests that included metabolic analysis, quantification of viable cells (CFU/mL), and phospholipase activity evaluation of mono-species and dual-species biofilms of *C. albicans* and *E. faecalis* developed for 7 days. Each laboratory phase was performed independently, in duplicate, considering *n* = 8/group.

### 2.2. Microorganisms Involved

Reference *C. albicans* (ATCC 90028) and *E. faecalis* (ATCC 29212) strains were used. The fungal strain was reactivated in Agar Sabouraud Dextrose culture medium (Difco, Detroit, United States) and incubated at 37°C for 24 h, aerobically. After this time, three to five colonies were collected and suspended in 5 mL of RPMI 1640 broth (Sigma Aldrich, St. Louis, MO, United States). After incubation at 37°C for 24 h, the set was centrifuged (Centrifuge Excelsa 205 N, FANEM LTDA., Brazil) and the cells suspended in sterile saline (NaCl 0.9% w/v). The *E. faecalis* (ATCC 29212) reactivation was carried out in BHI agar culture medium (Sigma Aldrich, St. Louis, MO, United States), and the colonies, after cultivation, were resuspended in BHI broth. All incubation and temperature parameters were the same as those performed for *C. albicans* [15].

### 2.3. Preparation of Phytoconstituents

Phytoconstituents cinnamaldehyde and *α*-terpineol were bought from the company Sigma-Aldrich (Saint Louis, United States) with the technical specifications shown in Table 1. Considering the molecular weight substances, a 10 mg/mL (1%) concentration was calculated, which was diluted in the RPMI 1640 medium and 0.01% (*v*/*v*) of Tween 80 was used only as an emulsifier in both solutions [16].

In addition, 2.5% NaOCl (v/v) solutions were used and 2% CHX (*v*/*v*), as antimicrobial controls (Dilecta, Brazil). The culture medium without the addition of any antimicrobial solution was used as a growth control in all stages of the experiments.

The concentration of phytoconstituents related is based on studies reported in the literature [16, 17] and previous studies (unpublished data). The NaOCl and chlorhexidine concentrations are commonly used in clinical endodontic treatments.

### 2.4. *Candida albicans* and *Enterococcus faecalis* Biofilms Mono-Species and Dual-Species

The cell concentration was determined in a spectrophotometer, at 600-nm wavelength. The cell density established at the absorbance of 0.1 (LGL Scientific 0741/16, Brazil) was performed for each microorganism to establish a 1:1 ratio of each species in culture, corresponding to concentrations of 1.0 × 10^8^ CFU/mL for *E. faecalis* and 1.0 × 10^6^ CFU/mL for *C. albicans* [18]. The RPMI 1640 medium was used to prepare the inoculum in biofilm assays.

After the *inoculum* preparation, both mono-species and dual-species biofilms showed the same initial cell density for the mentioned microorganisms. To prepare the *inoculum*, the initial concentration of each microorganism was considered, adjusted to a final volume of 300 *μ*L for each compartment on the 96-well plates (Kasvi). For dual-species biofilms, both *C. albicans* and *E. faecalis* were added to the *inoculum* [19]. Dual-species biofilm experiments were performed using a 1:1 ratio of *E. faecalis* to *C. albicans* cells to seed the biofilms that each species could be equally represented irrespective of their cell size differences. Biofilms samples were cultured for 7 days, with medium change every 48 h, without new cell addition. The samples were incubated in microaerophilia (anaerobic jar candle technique) at 37°C.

### 2.5. Exposure to Phytoconstituents

After the time culture (7 days), biofilms were exposed to phytoconstituents for 24 h additional, at 10 mg/mL concentration. Then, 7 days was selected as the maturation period for the biofilm to closely replicate the microenvironment typical of chronic endodontic infections [20]. Therefore, we established a maximum exposure time of 24 h to assess the antimicrobial effects of the substances on a mature biofilm, recognizing that this duration may not be clinically applicable for use as an auxiliary chemical irrigants [21]. The phytoconstituents were diluted (1:2), with 300 *μ*L being added to each compartment and the samples incubated at 37°C. After an additional 24 h, the analyses were performed. The control substances were also diluted in the medium and placed over the samples following the same parameters as the test substances. Sterility controls were maintained.

### 2.6. Cellular Metabolism Evaluation

After a 24-h exposure to phytoconstituents, the metabolic activity of the biofilms was evaluated using the methyl tetrazolium (MTT) salt assay. The culture medium was discarded, and 250 *μ*L of the fresh culture medium containing 10% MTT salt was added before incubating the system at 37°C for 3 h. No washing of the compartments was performed before the addition of MTT [22]. The salt was oxidized by the enzyme succinic dehydrogenase (SDH), allowing for the observation of viable cell metabolism. Subsequently, the medium was removed, and 250 *μ*L of acid isopropanol (6N-HCl) was added. The samples were then homogenized using a Global Trade Technology GT-20IBDU. From this, 100 *μ*L of the mixture was transferred in duplicate to 96-well plates, and the absorbance was measured at 560 nm [15].

### 2.7. Colony-Forming Unit Count (CFU/mL)

To collect biofilm samples and colony-forming units/mL counting, the entire contents were removed from the 24-well plate compartments. Then, 100 *μ*L of sterile saline solution was inserted into each well and the samples released by the scraper technique. Subsequently, 20 *μ*L of the microbial suspension was transferred into microplates containing 180 *μ*L of saline solution and subjected to serial dilutions (10^−2^ to 10^−7^) to assess the viable microorganism count. Then, 10-*μ*L aliquots were seeded on Sabouraud Dextrose Agar plates containing chloramphenicol, for counting *C. albicans*, in triplicate, corresponding to each serial dilution and the plates incubated at 37° C for 24 h. For counting *E. faecalis*, BHI agar was used. Viable cells were counted in each selective medium, and the values multiplied by serial dilution and converted into logarithmic scale [18].

### 2.8. Phospholipase Activity

The phospholipase activity evaluation was carried out as reported by Price et al. [23] with some modifications. The medium base was prepared using 2 g of tryptone, 6 g of glucose, 11.46 g of sodium chloride, 0.11 g of calcium chloride, and 4 g of agar for 200-mL distilled water. After sterilization in an autoclave, the medium was cooled down to 50°C and 15 mL of egg yolk emulsion inserted with 0.15% potassium tellurite (Laborclin Products Pinhais, PR, Brazil). After mono-species and dual-species biofilm cultivation, the entire contents of the plate were removed, 300 *μ*L of sterile saline was added, and the samples were homogenized. Subsequently, 10-*μ*L aliquots from each well, in duplicate, were inoculated into the culture medium based on egg yolk for 7 days at 37°C. The experiment to evaluate phospholipase activity was carried out only for *C. albicans* mono-species and dual-species biofilm.

The lipid substrate present in the egg yolk hydrolysis results in a calcium complex with fatty acid formation expelled by the action of secretory enzymes, resulting in a precipitation zone around the colony; thus, after the period of 7 days, the surrounding hyaline zone colonies were measured in mm. Phospholipase activity (PZ-PL value) was measured in terms of total colony diameter and the precipitation zone ratio. The activity was categorized into 3 scores: PZ − PL < 0.64 (very high), 0.64 < PZ − PL < 1 (high), and PZ − PL = 1 (negative) [24].

### 2.9. Data Analysis

Data obtained from the experiments were analyzed statistically as numerical variables, considering their interspace or continuous behavior. Initially, normality was verified by Shapiro–Wilk test, as well as homoscedasticity by Levene *F* test. The inferential analysis was performed with SPSS for Windows, version 19.0 statistical program, using Kruskal–Wallis and stepwise with adjusted Bonferroni for nonnormal data and ANOVA one-way tests complemented by Tukey for normal data (*α* = 5%).

## 3. Results

For all the assays evaluating cell metabolism, cell viability, and phospholipase activity, sterility control was rigorously maintained, ensuring the absence of contamination. The blank control was confirmed to exhibit no microbial growth, corresponding to a value of zero.

**Figure 1:**
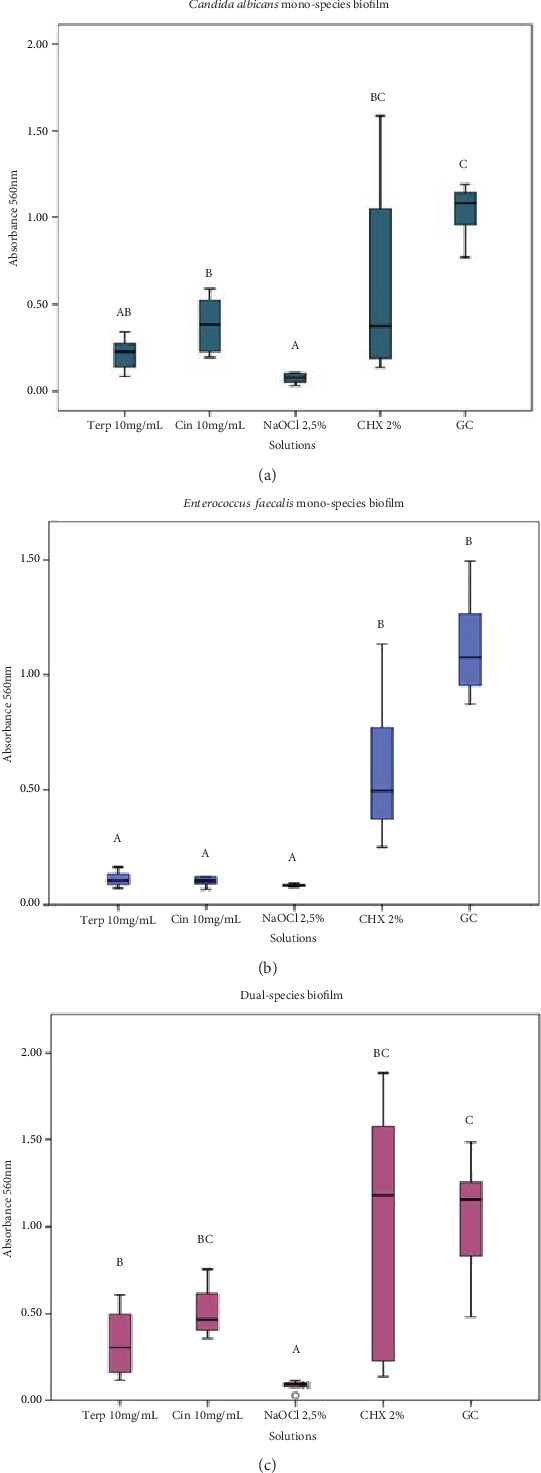
Cellular metabolism assessed by MTT test. (a) *Candida albicans* mono-species biofilm cellular metabolism. (b) *Enterococcus faecalis* mono-species biofilm cellular metabolism. (c) Dual-species of *Enterococcus faecalis* and *Candida albicans* biofilm cellular metabolism. Biofilms were formed for 7 days. Representation in absorbance (560 nm). ⁣^∗^Different letters determine statistical difference (Kruskal–Wallis, *p* < 0.05). Box diagram: each box contains 50% of the group data; the lower and upper limits of the boxes represent the 25 and 75 percentiles, respectively; the antennas ends represent the group minimum and maximum values; and the horizontal line inside the box represents the median.

Regarding the analysis of cellular metabolism using the MTT assay in *C. albicans* mono-species biofilms, *α*-terpineol at 10 mg/mL (mean absorbance = 0.215) did not show significant differences compared to cinnamaldehyde at 10 mg/mL (*p* > 0.05), 2.5% NaOCl (*p* > 0.05), or 2% chlorhexidine (*p* > 0.05), exhibiting significant differences only about the growth control (*p* < 0.0001). In contrast, cinnamaldehyde (mean absorbance = 0.384) did not differ significantly from *α*-terpineol or chlorhexidine (*p* > 0.05) but demonstrated significant differences compared to the growth control (*p* < 0.05) and NaOCl (*p* < 0.0001) (Figure 1a).

For the *E. faecalis* mono-species biofilm, *α*-terpineol (mean absorbance = 0.111) did not exhibit significant differences compared to NaOCl (*p* > 0.05) but showed significant differences relative to chlorhexidine (*p* < 0.0001) and the growth control (*p* < 0.0001). Cinnamaldehyde (mean absorbance = 0.121) demonstrated significant differences compared to chlorhexidine (*p* < 0.05) and the growth control (*p* < 0.0001), while no significant differences were observed about NaOCl (*p* > 0.05) (Figure 1b). In the dual-species biofilm, only *α*-terpineol (mean absorbance = 0.121) and NaOCl exhibited significant differences compared to the growth control (*p* < 0.05), although they were not statistically similar to each other (Figure 1c). Cinnamaldehyde (mean absorbance = 0.502) did not show significant differences in comparison to the growth control (*p* > 0.05).

**Figure 2:**
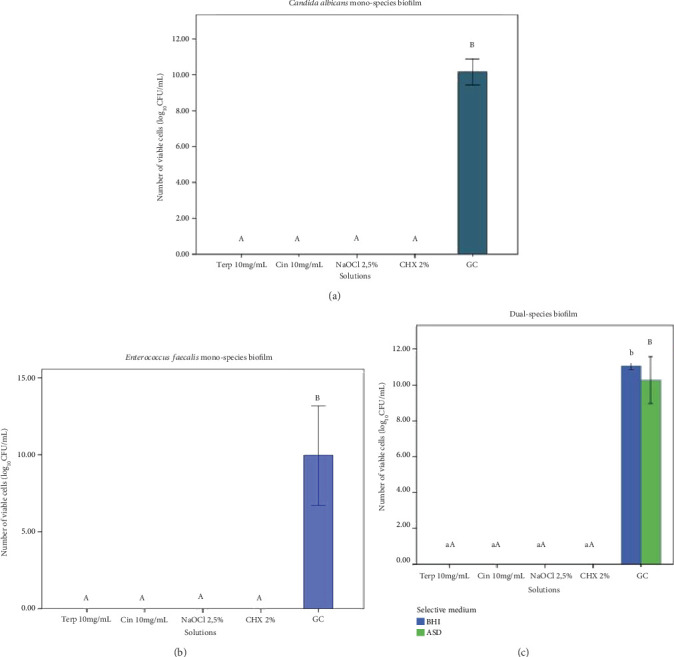
Cell viability assessed by CFU/mL. (a) *Candida albicans* mono-species biofilm cell viability. (b) *Enterococcus faecalis* mono-species biofilm cell viability. (c) Dual-species of *Enterococcus faecalis* and *Candida albicans* biofilm cell viability. Biofilms were formed for 7 days. Logarithmic representation base 10 (log_10_ UFC/mL). ⁣^∗^Different letters determine statistical difference (one-way ANOVA and Tukey, *p* < 0.05). Box diagram: each box represents group average and the antennas represent standard deviation.

Regarding cell viability analysis through colony-forming unit (CFU/mL) counts, cinnamaldehyde and *α*-terpineol did not differ significantly from NaOCl or chlorhexidine (*p* > 0.05) but showed significant differences when compared to the growth control (*p* < 0.05) in *C. albicans* mono-species biofilms (Figure 2a). The same pattern was observed for *E. faecalis* mono-species biofilms (Figure 2b), where the tested phytoconstituents exhibited significant differences in contrast to the growth control (*p* < 0.05). In the dual-species biofilm (Figure 2c), all tested substances exhibited significant differences compared to the growth control (*p* < 0.05) for both culture media evaluated over 7 days. The CFU/mL mean values ranged from 0.00 for the phytoconstituents and antimicrobial controls, both in mono-species and dual-species biofilms, to 10.14 for the growth control of *C. albicans* mono-species biofilms, 9.95 for *E. faecalis* mono-species biofilms, and 10.32 for the dual-species biofilm.

**Figure 3:**
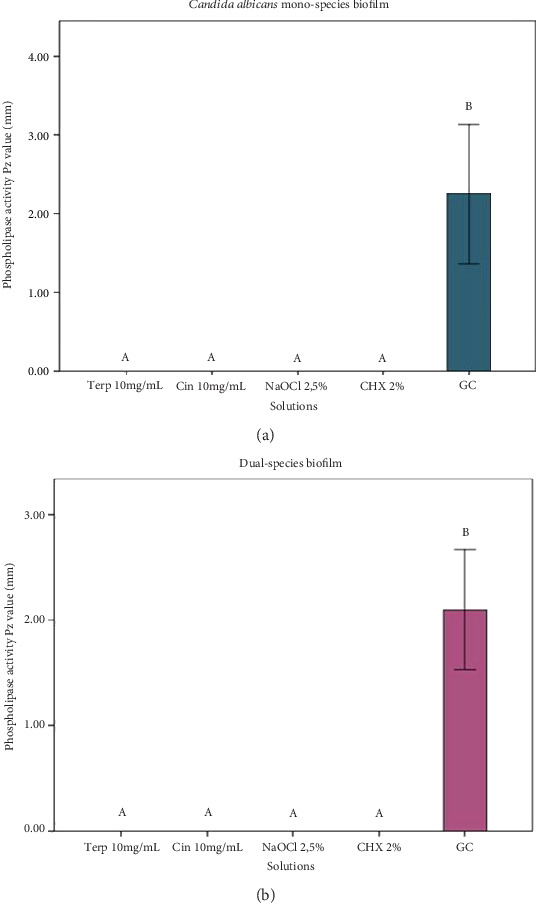
Phospholipase activity assessed by PZ value (nm). (a) *Candida albicans* mono-species biofilm phospholipase activity. (b) Dual-species of *Enterococcus faecalis* and *Candida albicans* biofilm phospholipase activity. Biofilms were formed for 7 days. ⁣^∗^Different letters determine statistical difference (one-way ANOVA and Tukey, *p* < 0.05). Box diagram: each box represents group average and antennas represent standard deviation.

In the analysis of phospholipase activity, the test solutions were effective against *C. albicans* in both mono-species and dual-species biofilms, with both showing significant differences compared to the growth control (*p* < 0.05) (Figure 3a,b). Phospholipase activity was not detected in the *E. faecalis* mono-species biofilm. No phospholipase activity was observed in biofilms treated with the tested phytoconstituents or antimicrobial controls. The average phospholipase activity in the growth controls of *C. albicans* mono-species and dual-species biofilms was 2.24 mm and 2.09 mm, respectively.

## 4. Discussion

Endodontic treatment success is based on the triad of disinfection: disinfection, instrumentation, and filling; therefore, it is necessary to completely remove microorganisms from within the canal system of the root [6], either through instrumentation, or auxiliary chemical solutions, or intracanal medication. Although the traditional auxiliary chemical substances used as irrigators have antimicrobial effects verified, their potential cytotoxic effects must be considered. For this reason, studies seek natural substances with antimicrobial properties and reduced adverse effects that can be used as endodontic irrigators [25, 26].

Thus, the present study analyzed the antimicrobial activity of phytoconstituents cinnamaldehyde and *α*-terpineol. Cinnamaldehyde is the main component of cinnamon extract (*Cinnamomum cassia*) and its mechanism of action is dose-dependent, based on the disorganization of the cell membrane, affecting its permeability, through the reduction of ergosterol biosynthesis [27, 28]. This present study observed cinnamaldehyde inhibitory efficacy on the used microorganisms' cell viability, corroborating to its mechanism of action. In addition, cinnamaldehyde can inhibit adhesion, morphological transition, and biofilms formation through increased farnesol secretion induced by Dpp3 expression, influencing biofilm virulence [29]. The ability of adhesion and formation of the biofilm and the morphological transition directly influence the virulence of the biofilm, corroborating with our data found of decreased cellular metabolism of biofilms by cinnamaldehyde, which can be suggested that influenced the virulence of biofilms.

*α*-Terpineol is a tea tree (*Melaleuca alternifolia*) active component, which has a hydroxyl group in its chemical composition, making it soluble in water. Its antimicrobial action is related to this solubility, which allows the oil to enter, destabilizing the plasma membrane, through an osmotic shock [30, 31]. *α*-Terpineol can directly induce irreversible morphological and structural changes in the cell, resulting in death [31]. The antimicrobial effect of the phytoconstituent is dose–time-dependent [30].

It was observed that *C. albicans* and *E. faecalis* mono-species biofilm cellular metabolism was reduced by cinnamaldehyde and *α*-terpineol. However, in dual-species biofilm, cinnamaldehyde showed limited antimicrobial activity, suggesting that the increased structural complexity of these biofilms confers more resistance to antimicrobial agents, necessitating higher concentrations for similar efficacy.

Attention is drawn to the chlorhexidine results in MTT test, where in all evaluated biofilms, despite its antimicrobial nature already established in the literature, presented results like growth control. It is suggested that the precipitate formed by chlorhexidine at the bottom of the compartments acted by interfering in absorbance analysis, as it reduces the light passage, leading to a false negative result. When NaOCl comes into contact with CHX, a brown precipitate is formed [32], which, comparing with our results, we can infer that this precipitate would be the product of chlorhexidine solubilization.

In addition, it was possible to observe a reduction in biofilm cell viability exposed to phytoconstituents, being comparable to positive controls, including chlorhexidine, which in this analysis did not present false-negative results. In colony-forming unit analysis, the precipitate formed by chlorhexidine does not interfere with the results, which confirms its antimicrobial effect.

Our findings also underscore the differential efficacy of cinnamaldehyde and *α*-terpineol across distinct assay methodologies. The MTT assay, which assesses cellular metabolic activity, may yield false positives by detecting apoptotic cells as viable due to their continued ability to reduce the tetrazolium compound. In contrast, the colony-forming unit (CFU) assay provides a more direct quantification of viable cell numbers, offering a more accurate representation of antimicrobial efficacy. These methodological variations highlight the necessity of accounting for assay-specific factors when interpreting the results of antimicrobial studies. The discrepancies observed between CFU and MTT assay results align with findings in the literature [21], which attribute these differences to the minimum cell density threshold required for sufficient enzymatic reduction in tetrazolium-based assays.

Studies involving the use of cinnamaldehyde in biofilms of *C. albicans* and *E. faecalis* show an inhibitory concentration lower than that used by us. However, it is worth mentioning that there is a difference in the time of maturation and development of biofilms. Most studies evaluated the inhibitory effect of cinnamaldehyde on 24-h biofilms [14, 33, 34], we evaluated it on a 7-day biofilm, so we overestimated the dose of cinnamaldehyde, suggesting that a 7-day biofilm is more developed and showing that it is more difficult for antimicrobial agents to achieve the desired effect. Furthermore, the presence of a greater quantity of extracellular matrix in biofilms cultured for 7 days must be considered, determining a greater challenge to the antimicrobial. Consistent with the concentration used in our study, 1% cinnamaldehyde effectively inhibited *E. faecalis* biofilm regrowth after 10 days of treatment. In contrast, despite the known substantivity of chlorhexidine, an initial biofilm recovery was observed [21].

Regarding *α*-terpineol, its antimicrobial effects against oral pathogens, such as *C. albicans* and *Streptococcus mutans*, have been previously reported [17, 30, 35]. The minimum inhibitory concentration (MIC) determined for *C. albicans* was 0.25% [30], while for *Streptococcus mutans*, it was 0.8 mg/mL [35]. It is important to note that these studies were conducted in 24-h cultures and mono-species biofilms, which may influence the effective inhibitory concentration. Furthermore, the concentration required to inhibit planktonic cultures cannot be directly compared to that needed for biofilm inhibition. Biofilms exhibit complex and multifactorial resistance mechanisms, including genetic adaptation and limited diffusion through the biofilm matrix, which hinder antimicrobial action [12]. Additionally, studies assessing the effects of *α*-terpineol on oral biofilms are scarce, and there are no reports in the literature specifically addressing its impact on *E. faecalis* biofilms. Another relevant aspect is the lack of studies investigating the effects of *α*-terpineol on cocultures of fungi and bacteria, particularly *C. albicans* and *E. faecalis*.

Another parameter evaluated in this study was phospholipase activity. Extracellular phospholipases facilitate hyphal penetration and induce cell lysis by interacting with the plasma membrane, thus contributing to *C. albicans* virulence [36]. *C. albicans* is known to be a major producer of extracellular enzymes, including phospholipases and proteinases [37]. Cinnamaldehyde has been shown to reduce phospholipase activity in *C. albicans* in a dose-dependent manner [38]. In the present study, both cinnamaldehyde and *α*-terpineol demonstrated the ability to inhibit *C. albicans* mono-species biofilms and dual-species phospholipase activity. However, the assay was not performed for *E. faecalis* single-species biofilms, as this microorganism does not exhibit affinity for the medium used to evaluate phospholipase activity.

The selection of the *E. faecalis* strain is a critical consideration, as ATCC 29212 is isolated from the urinary tract, whereas ATCC 4083 originates from the root canal. Given the influence of strain resistance on antimicrobial efficacy, ATCC 29212 has been shown to exhibit greater resistance to calcium hydroxide and 2% chlorhexidine compared to ATCC 4083 [39]. In this study, the more resistant ATCC 29212 strain was selected, ensuring that the antimicrobial activity of the tested phytoconstituents was assessed against a higher resistance benchmark.

Thus, based on the results obtained in the study, cinnamaldehyde and *α*-terpineol exhibit potential antimicrobial activity against *C. albicans* and *E. faecalis* in both mono- and dual-species biofilms. It is important to note that the concentrations used exceeded the MICs reported in the literature. However, due to the lack of established parameters for these phytoconstituents in 7-day biofilms, a higher concentration was intentionally selected to establish an upper threshold for inhibiting mature biofilms, considering the highly specific environmental conditions of the endodontic system. Moreover, considering that endodontic treatment relies on both chemical and mechanical approaches, this study exclusively evaluated the chemical method, necessitating an intentional overestimation of treatment duration for antimicrobial assessment. Once the effects of these substances on biofilms are established, future studies can be designed to develop a protocol integrating mechanical instrumentation while optimizing the exposure time of the chemical agent to reflect clinical treatment conditions.

This study represents an initial in vitro investigation into the antimicrobial activity of cinnamaldehyde and *α*-terpineol against dual-species biofilms involved in endodontic infections, marking a foundational step toward their potential clinical application. The study about new approaches in the endodontics field has been frequent nowadays, especially regarding the use of other molecules with biological effects. Although NaOCl and CHX have already established protocols for use, there are adverse effects for the use of both [7, 8]. The search for molecules with greater tolerability and greater bioavailability is encouraged.

These molecules are potential biological agents that can present antioxidant and anti-inflammatory effects, dependent on concentration, in addition to antimicrobial activity [40, 41]. Neither NaOCl nor chlorhexidine presents these characteristics, which are also necessary during endodontic treatment. Although this is an experimental in vitro study, the literature indicates these effects are pertinent to these molecules.

The 10 mg/mL (1%) concentration used in this study was selected based on unpublished MIC data against *S. mutans* and *C. albicans* biofilms, considering mature endodontic biofilms' greater complexity and resistance. This clinically relevant concentration aligns with those of commonly used irrigants such as NaOCl (2.5%) and chlorhexidine (2%) and was applied without advanced delivery systems. Although effective, future studies will focus on cytotoxicity assessment and on optimizing delivery through functionalized systems to potentially reduce the required concentration.

Then, the integration of these phytoconstituents into endodontic practice necessitates further research to assess their efficacy within the complex anatomical and microbiological environment of the root canal, as well as to evaluate their cytotoxic and anti-inflammatory effects on host tissues. Future studies should focus on their activity against more complex, multispecies biofilms that better replicate endodontic infections. Additionally, comprehensive dose- and time-dependent analyses, along with cytotoxicity assessments in relevant cell lines, are crucial for determining safe and effective concentrations to support their clinical translation. Further laboratory investigations should also evaluate the effects of these phytoconstituents within the root canal system in combination with mechanical instrumentation, simulating real clinical conditions.

## 5. Conclusion

Cinnamaldehyde and *α*-terpineol at a concentration of 10 mg/mL demonstrated a reduction in colony-forming unit count, cellular metabolism, and phospholipase activity in *C. albicans* and *E. faecalis* in vitro biofilms. These findings suggest their potential as alternative antimicrobial molecules potentially applied in the endodontic field. However, further studies are required to confirm their efficacy and safety before clinical application.

## Figures and Tables

**Table 1:** Phytoconstituents selected for microbiological tests.

**Phytoconstituents**	**Molecular formula**	**Molecular weight**
Cinnamaldehyde (cin)	C_9_H_8_O	132.16 g/mol
*α*-Terpineol (terp)	C_10_H_18_O	154.25 g/mol

## Data Availability

The data generated and analyzed during this study are available from the corresponding authors upon reasonable request. The results of the data analysis have been incorporated into the article in the form of text, figures, and tables. Do not hesitate to contact the authors directly for any questions or further clarification.
